# Fringe Controls Naïve CD4^+^T Cells Differentiation through Modulating Notch Signaling in Asthmatic Rat Models

**DOI:** 10.1371/journal.pone.0047288

**Published:** 2012-10-10

**Authors:** Wen Gu, Weiguo Xu, Tao Ding, Xuejun Guo

**Affiliations:** Department of Respiratory Medicine, Xinhua Hospital, Shanghai Jiaotong University, School of Medicine, Shanghai, China; Fudan University, China

## Abstract

The ability of Notch signaling to regulate T helper cell development and differentiation has been widely accepted. Fringe, *O-fucose-β1,3-N*-acetylglucosaminyltransferases modulate Notch receptor expression and promote the Notch signaling pathway through receptor-ligand binding. In this study, we assayed the expression levels of three Fringe homologs in naive CD4^+^T cells in asthmatic rats. We found that Radical Fringe (Rfng) was highly expressed, whereas both Lunatic Fringe (Lfng) and Manic Fringe (Mfng) were expressed at low levels. Down-regulation of Rfng using siRNA, and overexpression of Lfng or Mfng enhanced Th1 subset lineages and diminished Th2 subset lineages. Notch signaling was more activated in asthmatic naïve CD4^+^T cells than in control cells, and Lfng, but not Mfng or Rfng, partly inhibited Notch signaling in asthmatic naïve CD4^+^T lymphocytes. Lfng overexpression resulted in significantly decreased Th2 cytokine production in asthma, which was the same effect as the GSI (γ-secretase inhibitor) treatment alone, but had an increased effect on Th1 cytokines than GSI treatment. Collectively, these data identify the essential role of Fringe modulating naïve CD4^+^T cells differentiation through Notch signaling. Lfng regulated Th2 cells differentiation via a Notch-dependent manner and Th1 cells differentiation via a Notch-independent manner. Fringe could be a therapeutic strategy for the management and prevention of allergic asthma.

## Introduction

Asthma is a complex disease characterized by persistent airway inflammation and airway hyperresponsiveness (AHR) [1]. In asthmatics, allergen-specific IgE is elevated and the Th2 cytokines IL-4, -5, -9 and -13 are secreted by T-helper cells (Th), followed by subsequent activation of mast cells, infiltration of eosinophils and airway smooth muscle constriction [2]. After antigenic stimulation, naïve CD4^+^T cells differentiate into two distinct helper T cell subsets, Th1 and Th2 cells [3]. It has been generally accepted that disturbance of Th1 and Th2 plays an important role in the pathogenesis of asthma [4], [5], highlighting the importance of T-helper cell (CD4^+^) activation and differentiation in asthma.

The Notch signaling pathway is an evolutionarily highly conserved pathway that regulates a broad range of cell fate decisions by functioning in many developmental processes, such as embryogenesis, neurogenesis and lymphopoiesis [6], [7], [8]. Recent evidence has highlighted the role of Notch pathways in peripheral Th cell activation and differentiation [6], [9]. Interference with Notch signaling by transgenic expression of Notch modulators or blocking Notch by γ-secretase inhibitor also blocks T cell development [10], [11], [12].

Notch receptors and their ligands are expressed on the surface of mature lymphocytes and APCs, respectively. Notch signaling activated through receptor-ligands promote Th cell differentiation in response to different Th1-or Th2-promoting stimuli [13], [14], [15], [16].

In vertebrates, there are four Notch receptors (Notch1-4) and five Notch ligands, the Delta-like families (Delta1, Delta3 and Delta4) and Jagged families (Jagged1 and Jagged2) [17], [18]. After the Notch receptor binds a ligand, it releases its intracellular domain into the cytoplasm, translocates into the nucleus and induces the transcriptional activation of the Hes family [19], [20].

Notch signaling is modulated by several proteins [21]. One of those modulators, Fringe, has glycosyltransferase activity, attaching (GlcNAc) *N*-acetylglucosamine to *O*-Fucose on the extracellular domain of Notch [22]. Fringe is a highly conserved, Golgi-localized glycosyltransferase that critically influences the Notch receptor-ligand interactions essential for normal Notch signaling [23], [24]. In fruit flies, the glycosylation by fringe enhances Delta-stimulated Notch signaling, while it inhibits Serrate-stimulated Notch signaling [25]. In vertebrates, three fringe family genes have been identified and designated as lunatic fringe, manic fringe and radical fringe [26]. The effects of these mammalian fringes on Notch have been controversial. Yang and colleagues showed complex receptor-ligand interactions [27]. Tsukumo and colleagues showed that Lunatic fringe enhanced T cell differentiation through Notch signaling [28]. Okamoto and colleagues showed the essential role of receptor-ligand interactions in allergic airway inflammation [29]. However, questions remain as to the exact roles of Notch signaling in Th cell differentiation.

Our team focused on Fringe and its role in Th cell lineage commitment in allergic airway diseases. In this study, we showed that Lunatic fringe (Lfng) and Manic fringe (Mfng) were down regulated in naïve CD4^+^T cells and that Radical fringe (Rfng) was up-regulated in OVA-sensitized asthmatic rat models. We further explored the relationship between Fringe and Notch through signaling transduction. We demonstrated that overexpression of Lfng (or Mfng) on naïve CD4^+^T cells using Lfng plasmids (or Mfng plasmids) and inhibition of Rfng expression using small interfering RNA (siRNA) prevented Th2 cytokine production and promoted Th1 cytokine production. Lfng played its role through a Notch signaling dependent manner. We come to the conclusion that Fringe may be a unique therapeutic opportunity for allergic airway diseases.

## Materials and Methods

### OVA-sensitized Chronic Airway Inflammation

Six- to eight-week-old male Winstar rats (mean weight 160 g±5 g) were kept under specific pathogen-free conditions. The animals were divided into two groups. The asthmatic group was sensitized with 2mg of OVA (grade V; Sigma-Aldrich, St. Louis, MO) and 200 mg of Alum in 1 ml 0.9% NaCl applied subcutaneously. As a second adjuvant, concentrated preparations of 5×10^9^ heat-killed *Bordetella pertussis* bacilli were given intraperitoneally at the same time. All sensitization procedures were repeated on day 7 after the first sensitization. Additionally, the rats were exposed to a challenge with an aerosol of 2% OVA at 0.25L/min for 30 min daily from day 14 to day 28. The control group was given NaCl on the same schedule. The rats were killed 2 days after the last OVA challenge, and the BAL (Bronchoalveolar Lavage) fluid and lungs were collected. All animal research was approved by the Shanghai Jiaotong University School of Medicine Animal Care and Use Committee.

### Assessment of Airway Function

Airway function was assessed by measuring changes in lung resistance (R_L_) and dynamic compliance (Cdyn) in response to increasing doses of inhaled methacholine (Mch) (Buxco Biosystem, Amercia). Data are expressed as percentage change from baseline R_L_ values obtained after inhalation of saline. The baseline R_L_ responses to saline in the individual groups were not significantly different.

### Bronchoalveolar Lavage(BAL)

BAL was performed by intratracheal insertion of catheter and lavaging with 5 ml of cold PBS. The fluid was retrieved by gentle aspiration, and this procedure was repeated 10 times. The BAL fluid was pooled and centrifuged (400×g, 10 min). The supernatants were collected, and the cell pellet was resuspended in 1 ml of PBS.

### Preparation of Naïve CD4^+^T Cells

The chest cavity of each rat was opened using surgical dissection, and the inferior vena cava and abdominal aorta were clamped. The left atrium was opened by incision, and the right ventricle was infused with PBS to remove any residual blood in the pulmonary vasculature. The lung was cut into small pieces and was digested for 3 hr at 37°C with collagenase I (1 mg/ml; Invitrogen) and DNase (0.2 mg/ml, Invitrogen) in complete medium. The lung was further disrupted by aspiration through a 75 µm nylon mesh and lung cells were collected after centrifugation (300×g, 10 min). After being washed with PBS, mononuclear cells were isolated by Histopaque gradient centrifugation (Sigma-Aldrich). The cells were then subjected to positive selection with anti-CD4 magnetic beads on MS-positive selection columns (Miltenyi Biotech, Bergisch Gladbach, Germany) according to the manufacturer’s instructions. Then pooled CD4+T cells from 2–3 rats were stained with a biotin conjugated cocktail of anti-CD25, anti-CD44, anti-CD69, anti-CD45RO (ebioscience, San Diego, CA; Multiscience, CHN). After using biotin coupled beads, naïve CD4^+^T cells purification were done by negative selection on magnetic columns according to manufacturer’s protocols (Miltenyi Biotech, Bergisch Gladbach, Germany). Naïve CD4^+^T cells were stained with antibody CD3, CD4, CD25, CD69, CD45RA and CD45RO for flow cytometry analysis and the purity of them exceeded 90% (see Fig. S1). Isolated naïve CD4^+^T cells were seeded at 1×10^6^ cells/well in 24-well culture plates in complete medium (RPMI 1640 containing 10% heat-inactivated FCS, 100 U/ml penicillin, 100 µg/ml streptomycin, 2 mM l-glutamine, and 50 µm 2-ME) in a humidified atmosphere at 37°C in 5% CO_2_.

### Quantitative PCR (Q-PCR)

Total RNA was isolated from 3×10^6^ asthmatic group or control group cells in 24-well culture plates with Trizol Reagent (Invitrogen Life Technologies), followed by reverse transcription to cDNA (Takara). The amplification of cDNA was performed using SYBR premix EX Taq™ (Takara). The PCR protocol consisted of 95°C for 30 sec, followed by 40 cycles of 95°C for 5 sec and 60°C for 34 sec, with a final dissociation stage, and was performed with a ABI 7500 real-time PCR system (Applied Biosystems, Foster City, CA). We assumed that the amplification efficiency of the target and reference are approximately equal. The Ct of target genes was normalized to GAPDH (△Ct). Relative quantification and calculation were performed using the comparative threshold cycle method (2^−△△Ct^). The PCR primers are listed in Table 1.

**Table 1:** Summary of primer used for realtime PCR.

Gene	Primer sequence(5′-3′)	product
**Rfng**	Fwd 5′- TGCTGTTGCGTACCTGGATCTC-3′Rev 5′- ACAGCGGAACAATTGGTGTTGA-3′	**121** **bp**
**Lfng**	Fwd 5′-TTCATCGCCGTCAAGACCAC-3′Rev 5′-GTGCTTGGCCAAAGCTTCATC-3′	**132** **bp**
**Mfng**	Fwd 5′-CCTGCAAGATGGCTGCAGAA-3′Rev 5′-TGCAGCTCAGAGGCGTGTATG-3′	**187** **bp**
**Notch1**	Fwd 5′-GTGCCTGTCTGAGGTCAACGA-3′Rev 5′-TGTCACAGTTTGTCCCACTCCA-3′	**119** **bp**
**Jagged1**	Fwd 5′-GTCGGGATTTGGTTAATGGTT-3′Rev 5′-GTGGGACACAGACACGGGAAT-3′	**154** **bp**
**Delta1**	Fwd 5′-GCTGGAGGTAGATGAGTGTGC-3′Rev 5′-CACAGACCTTGCCATAGAAGC-3′	**113** **bp**
**Gapdh**	Fwd 5′-GGCACAGTCAAGGCTGAGAATG-3′Rev 5′-ATGGTGGTGAAGACGCCAGTA-3′	**143** **bp**

Fwd: forward; Rev: Reverse.

### Western Blotting

Naïve CD4^+^T cells were lysed, denatured and protein quantified using the Bradford protein assay. Equal quantities of protein were loaded and electrophoresed on 10%(w/v) SDS-PAGE and then transferred to PVDF membranes. The membranes were blocked in Tris-buffered saline, 0.1% Tween20, and 5% milk, and then incubated with primary antibodies overnight and washed in Tris-buffered saline/0.1% Tween20. Secondary antibodies were diluted in blocking buffer and incubated with the membranes for 2 hours at room temperature. Finally, the membranes were incubated in ECL reagents (Pierce) and exposed to film. The following antibodies were used: 1∶1000 purified rabbit anti-rat Radical Fringe antibody, 1∶1000 purified rabbit anti-rat Lunatic Fringe antibody (Abcam, Cambridge, UK), 1∶1000 purified goat anti-rat Manic Fringe (Santa Cruz Biotechnology, Santa Cruz, UA), and 1∶5000 donkey anti-goat IgG-HRP, 1∶5000 goat anti-rabbit IgG-HRP (Jackson).

### CD4^+^T Cell Transfection

Isolated naïve CD4^+^T cells from the asthmatic group were transfected using the Amaxa Nucleofector System (Amaxa). Then 5×10^6^ cells were resuspended in 100 µl of the appropriate Amaxa solution and transfected with 5 µg of plasmid DNA (pEGFP-N1-Lfng/pEGFP-N1-Mfng) or a 100 nM final concentration of siRNA in accordance with the manufacturer’s protocols (U-14 or V-24, respectively). The cells were immediately transferred to 1 ml of CRPMI 1640 and seeded as required. Eight hours later, transfected CD4^+^T cells were washed and used in subsequent experiments.

The full-length Lunatic Fringe (Lfng) cDNA and Manic Fringe (Mfng) cDNA were amplified by PCR from rat splenocytes and cloned into pEGFP-N1. Culture cells were divided into three groups: plasmid group, NC group and blank group.

Rfng-specific siRNA was 21-nt-long duplexes designed and synthesized by Dharmacon. The sequences were as follows: sense 5′- CGAGUAUGACAAGUUCA UAtt-3′ and antisense 5′-UAUGAACUUGUCAUACUCCtt-3′. Scramble siRNAs, as a negative control, contained the same nucleotide content as the selected siRNAs but in a random sequence and with no calculated target gene specificity, as assessed by BLASTing against all the sequence databases. Culture cells were divided into four groups: siRNA group, NC group, mock group and blank group.

### Notch Signaling Assay

RBP-Jκ-responsible luciferase reporter vector (pGa981-6) was presented by Department of Medical Genetics and Developmental Biology, Fourth Military Medical University. The vector was cloned in six repeated EBNA2RE using the BamH I and Xho I sites before the firefly luciferase gene site. Neg-pGa981-6 was constructed by removing the EBNA2RE and cloned in an irrelative fragment which cannot combine with RBP-Jκ.

Naïve CD4^+^T lymphocytes were transfected with 1 µg pGa981-6 or neg-pGa981-6 and 0.1 µg PRL-TK Renilla luciferase vector (10∶1) (Promega, Madison, WI) by PolyJet Reagent (Signagen) according to the manufacturer’s instruction. The cells were transferred to 24-well plates and cultured for 48 hr. The Firefly and Renilla luciferase activities were determined in whole cell lysates using a dual luciferase assay system (Promega).

The Hes1 levels were examined by quantitative PCR and Western blotting among interference cells and their counterparts.

### Block Notch Signaling by γ-secretase Inhibitor

We further used γ-secretase inhibitor II (MW167) to block Notch signaling. One milligram of MW167 was dissolved in 141.7 µl of dimethyl sulfoxide (DMSO). Interference and uninterference cells were seeded in 24-well plates separately, and 2.0 µl of MW167 solution was added per well. 2.0 µl DMSO was added as control group.

### Flow Cytometric Analysis of Proliferation

In some experiments, purified naïve CD4^+^T cells were resuspended to 1×10^7^ cells/ml in PBS and labeled by 5-(and 6-) carboxyfluorescein diacetate succinimidyl ester (CFSE) (Molecular Probe) and CD69-FITC as described below. After incubation for 15 min at 37°C in 5% CO_2_, the cells were washed three times with 10 volumes of PBS and centrifuged at 300×g for 5 min. The labeled cells were then stimulated with 5 µg/ml soluble anti-CD3 (clone G4.18; eBioscience) alone, anti-CD3 plus 2 µg/ml soluble anti-CD28 (clone JJ319; eBioscience), PHA-M (10 ng/ml) or PBS alone and incubated for 3 days at 37°C in 5% CO_2_. The cells were then washed and resuspended in PBS supplemented with 1% FCS and 0.025% sodium azide and analyzed using flow cytometry (BD Biosciences, San Diego, CA). The acquired FACS data were analyzed with the CellQuest program (BD Biosciences).

### CD4^+^T Cell Stimulation Assay

The response of naïve CD4^+^T cells transfected with plasmids or siRNAs was assessed as follows. Interference and uninterference cells were restimulated with anti-CD3 (5 µg/ml) and anti-CD28 (2 µg/ml) as described above for another 3 days. Total cells were collected to assess the expression of Lfng, Mfng, and Rfng by quantitative PCR and Western blotting. The mRNA levels of IL-4, IL-5, IFN-γ, IL-12, T-bet, and GATA-3 were further detected. The supernatants were harvested and analyzed by ELISA as described below. All data are representative of at least three independent experiments conducted in triplicate. The PCR primers were listed in Table 2.

**Table 2:** Summary of primer used for realtime PCR.

Gene	Primer sequence(5′-3′)	product
**IL-4**	Fwd 5′-CCCACCTTGCTGTCACCCTGTTC-3′Rev 5′-TTCTCCGTGGTGTTCCTTGTTGC-3′	**92** **bp**
**IL-5**	Fwd 5′- AGCTCTGTTGACGAGCAATGA-3′Rev 5′- CCCTCGGACAGTTTGATTCTT-3′	**121** **bp**
**IL-2**	Fwd 5′- AATGGAATTTGGTCCACCGAGA-3′Rev 5′- ACCAGCCATGAGCAGGTGAAC-3′	**104** **bp**
**IFN-γ**	Fwd 5′- AGGCCATCAGCAACAACATAAGTG-3′Rev 5′- GACAGCTTTGTGCTGGATCTGTG-3′	**140** **bp**
**GATA-3**	Fwd 5′- GCAATGCCTGTGGGCTGTACTA-3′Rev 5′- AGACATCTTACGGTTTCGGGTCTG-3′	**95** **bp**
**T-bet**	Fwd 5′- CCTGGACCCAACTGTCAACTGC-3′Rev 5′- ACGCTCACTGCTCGGAACTCTG-3′	**120** **bp**

### Cytokine Measurement by ELISA

The cytokine levels in the BAL fluid and cell culture supernatants were measured by ELISA. IL-4, IL-5, IFN-γ and IL-12 (R&D Systems, Minneapolis, MN) ELISAs were performed according to the manufacturer’s directions.

### Statistics

Statistical analysis was performed using Graphpad Prism Software (San Diego, CA). The results were expressed as the mean±SEM. The *t* test was used to determine differences between two groups, and the Tukey-Kramer test was used for comparisons between multiple groups. The Mann-Whitney *U* test was used for nonparametric analysis. The *p* values for significance were set to 0.05 for all tests.

## Results

### Airway Inflammation and Hyperresponsiveness (AHR) of OVA-sensitized Asthmatic Rat Models

**Figure 1:**
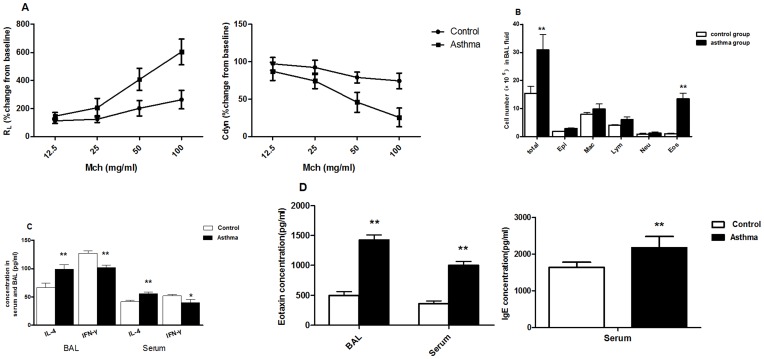
OVA-sensitized asthmatic rat models were established successfully. Airway function was detected and BAL fluid and serum were collected and evaluated by ELISA. **A**, R_L_ and Cdyn values were obtained in response to increasing concentrations of inhaled MCh, as described in material and methods. Data represented mean±SEM (n = 20 in each group). *p<0.05. **B**, Cellular composition of BAL fluid. **C**, Cytokine levels in BAL fluid and serum. **D**, Eotaxin levels in BAL fluid and serum. IgE levels in serum. Total, total cells; Epi, epithelium; Mac, macrophages; Lym, lymphocytes; Neu, neutrophils; Eos, eosinophils. Date represent the mean±SEM (n = 20 in each group). *p<0.05, **p<0.01 significant differences comparing asthmatic group and control group.

Lung resistance (R_L_) and dynamic compliance (Cdyn) values were obtained in response to increasing concentrations of inhaled Mch. There showed an increased lung resistance (R_L_) and decreased dynamic compliance (Cdyn) in OVA-sensitized asthmatic group than in control group (Fig. 1A).

The total cell numbers in BAL fluid were increased in the OVA-sensitized asthmatic group (30.95±5.24×10^5^ cells/ml; n = 20) compared with control group (15.47±2.42×10^5^ cells/ml; n = 20). In contrast, the asthmatic group developed significant eosinophilia (asthmatic group 13.44±2.02×10^5^ cells/ml vs. control group 1.02±0.19×10^5^ cells/ml; n = 20), with a smaller increase in BAL lymphocytes (Fig. 1B). The concentrations of IL-4, IFN-γ and Eotaxin in the serum and BAL were detected by ELISA as described above. In the asthmatic group, the Eotaxin and IL-4 levels increased significantly, while IFN-γ decreased significantly in both the serum and BAL. There was a significant increase in the serum IgE concentration (Fig. 1C, D).

**Figure 2:**
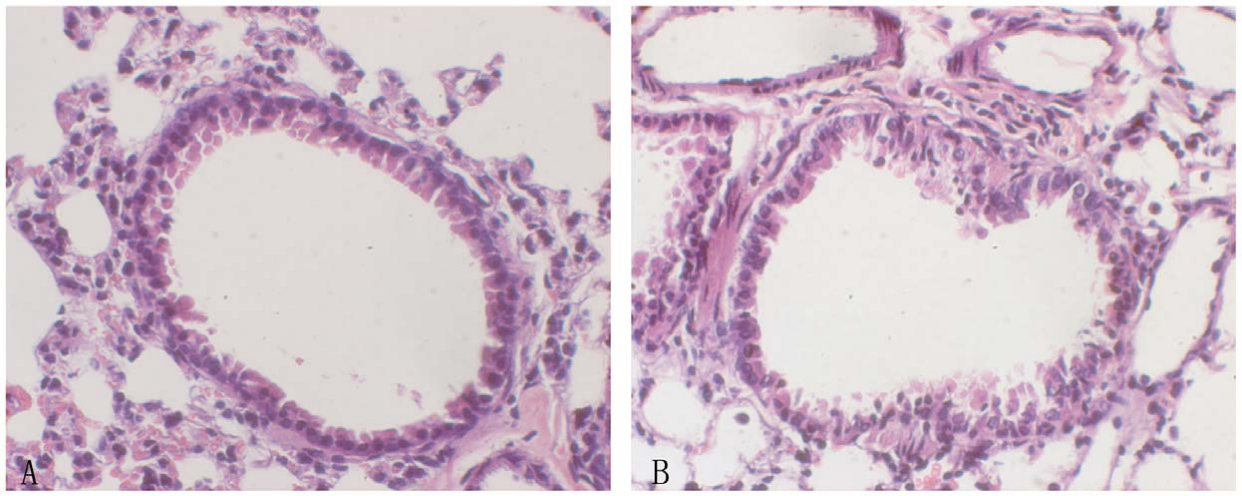
HE staining of lung tissue in OVA-sensitized asthmatic group and control group. **A**, control group; **B**, asthmatic group (Original magnification×400). Asthmatic group showed bronchial inflammation that exsisted in the forms of epithelial damage, wall thickening of the tunica mucosa bronchiorum, inflammatory cell infiltration into the lower layer of the mucous membrane and the surrounding bronchus, and accrementition and spasm of airway smooth muscle.

HE staining of asthmatic lung tissue showed bronchial inflammation that existed in the forms of epithelial damage, wall thickening of the tunica mucosa bronchiorum, inflammatory cell infiltration into the lower layer of the mucous membrane and the surrounding bronchus, and accrementition and spasm of airway smooth muscle (Fig. 2).

### Expression of Three Fringe Homologs on Naïve CD4^+^T Cells in Asthmatic Group

**Figure 3:**
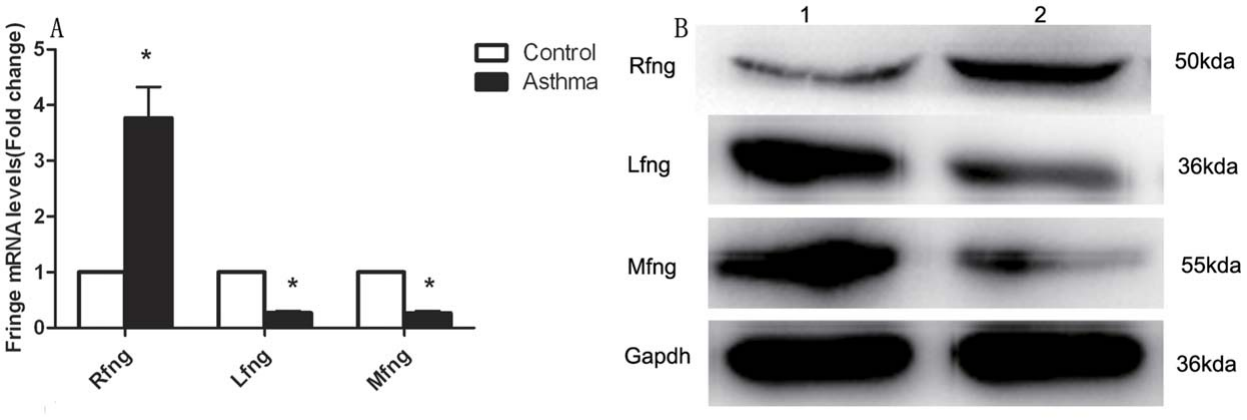
Expression of three Fringe homologes on naïve CD4^+^T lymphocytes. **A**, The mRNA expression levels of Rfng, Lfng and Mfng on naïve CD4^+^T lymphocytes were determined by Q-PCR, normalized to GAPDH. Results are from three independent experiments and the results for each group are expressed as mean±SEM. *P*<0.05, significant differences comparing asthmatic group and control group. **B**, Western blot was performed to detect the protein levels of Rfng, Lfng and Mfng (1, control group; 2, asthmatic group).

First we determined the endogenous expression patterns of three Fringe homologs on naïve CD4^+^T cells. Quantitative PCR analysis showed that CD4^+^T lymphocytes expressed all three types of Fringe (Rfng, Lfng, and Mfng). Compared with control group, Rfng mRNA increased more than 3-fold, whereas both Lfng and Mfng mRNA decreased approximately 30% in asthmatic group (Fig. 3A). Western blotting was used to detect the protein levels of three Fringe family genes. Rfng protein was expressed higher and Lfng or Mfng proteins were expressed lower in asthmatic CD4^+^T lymphocytes, which was consistent with the mRNA levels (Fig. 3B). Based on these analyses, we chose to develop Rfng targeted SiRNA and full-length Lfng or Mfng cDNA sequences cloned into pEGFP-N1 vectors for subsequent functional studies in CD4^+^T lymphocytes.

### Gene Silencing Treated with Rfng-specific siRNA and Overexpressing Lfng or Mfng in Asthmatic CD4^+^T Lymphocytes Efficiently

**Figure 4:**
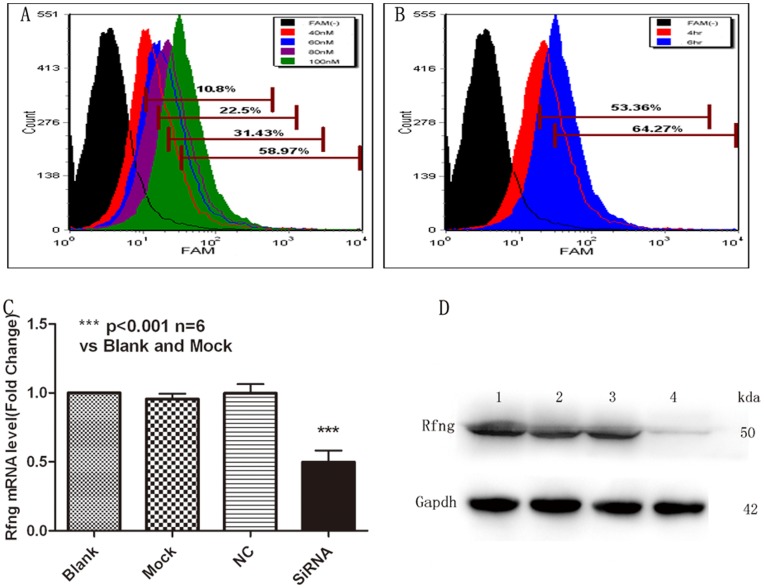
Asthmatic naïve CD4^+^T cells are efficiently transfected with SiRNA. CD4^+^T (5×10^6^) cells were transfected by Amaxa Nucleofection, protocol U-14, with 40 nM, 60 nM, 80 nM, 100 nM FAM-tagged SiRNA. The transfection conditions optimized by flow cytometry analysis. Q-PCR and Western blot were performed to determined the mRNA levels and protein levels after SiRNA-Rfng transfection. **A**, The optimal final concentration of SiRNA was 100nM (the MOI is 58.97%). **B**, The optimal time of transfection is more than 6 hr (64.27%). **C**, Real-time PCR analysis of Rfng levels in transfected naïve CD4^+^T cells. Blank-treated results were taken as 1. Results are from three independent experiments. The data for each group are expressed as means±SEM. *** *p*<0.05, significant differences between siRNA-Rfng and blank-treated, mock-treated or SiRNA-scrambled CD4^+^T cells. **D**, Rfng protein levels in transfected CD4^+^T cells. CD4^+^T cells were unmanipulated (blank, lane 1), transfected with reagent alone (mock, lane 2), siRNA scrambled (lane 3), or siRNA-Rfng (Lane 4). Transfected CD4^+^T cells were lysates and collected to assess the expression of Rfng by Western blot. Gapdh was used as a loading control. Representative of one of three similar experiments.

We constructed Rfng-specific siRNA and pEGFP-N1-Lfng vector (pEGFP-N1-Mfng vector) for further study and developed a protocol for introducing them into naïve CD4^+^T cells. We chose to use the Amaxa Nucleofection System for transfection. The siRNA transfection conditions for CD4^+^T cells were first optimized by flow cytometry detection (Fig. 4A, B). The optimal siRNA concentration for the siRNA group demonstrated a decrease in the expression levels of Rfng mRNA by approximately 50% (Fig. 4C). The protein levels also showed a significant reduction in the siRNA group compared with the mock group or the siRNA-scramble group (Fig. 4D).

**Figure 5:**
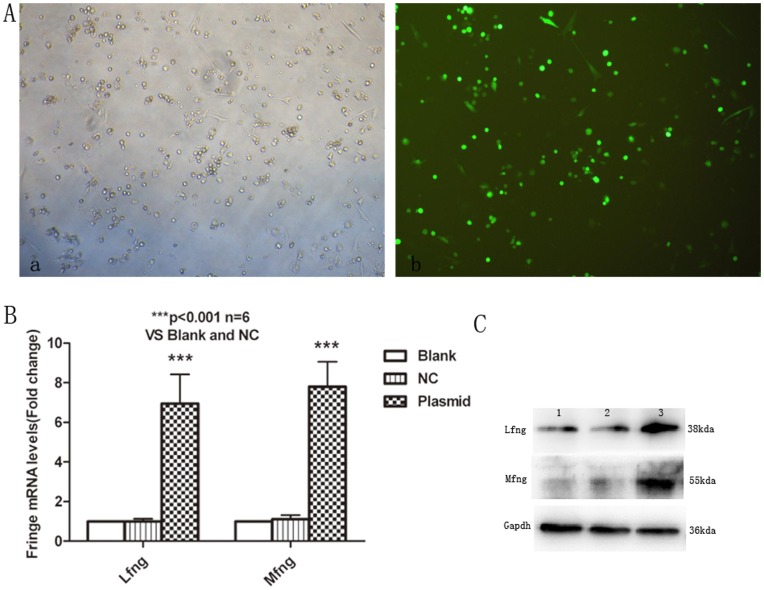
Overexpression of Lfng and Mfng in naïve CD4^+^T cells. A , Asthmatic naïve CD4^+^T cells were transfected with pEGFP-N1 plasimd using Amaxa Nucleofection System. 5×10^6^ cells were resuspended in 100 µl of the appropriate Amaxa solution and transfected with 5 µg pEGFP-N1 plasimd. 6–8 hrs’ later, GFP expression was detected under fluorescence microscope to optimize the transfection conditions. The optimal transfection efficiency was approximately 80% (a, bright field; b, fluorescence field). Original magnification was×100. **B**, Real-time PCR analysis of Lfng and Mfng levels in transfected CD4^+^T cells. Blank-treated results were taken as 1. Results are from three independent experiments. The data for each group are expressed as means±SEM. *** *p*<0.001, significant differences between plasmid overexpression group and blank group, or NC (pEGFP-N1) group CD4^+^T cells. **C,** Lfng and Mfng protein levels in transfected CD4^+^T cells. CD4^+^T cells were unmanipulated (Blank, lane 1), transfected with pEGFP-N1 plasmid (NC, lane 2), or transfected with Lfng plasmid or Mfng plasmid (Lane 4). Representative of one of three similar experiments.

Asthmatic CD4^+^T cells were transfected with pEGFP-N1-Lfng vector pEGFP-N1-Mfng using the Amaxa Nucleofection System. The transfection conditions were optimized by GFP expression under a fluorescence microscope (Fig. 5A). The expression levels of Lfng (Mfng) mRNA and the protein levels in naïve CD4^+^T cells were analyzed by real-time PCR and Western blot, respectively. In naïve CD4^+^T cells, the levels of Lfng mRNA increased 7-fold and Mfng mRNA increased 8-fold compared with those transfected with pEGFP-N1 alone (Fig. 5B). The protein levels of Lfng or Mfng were also markedly reduced (Fig. 5C).

### Down-regulation of Rfng and Overexpression of Lfng or Mfng Enhanced Th1 Cytokines but Suppressed Th2 Cytokine Production

**Figure 6:**
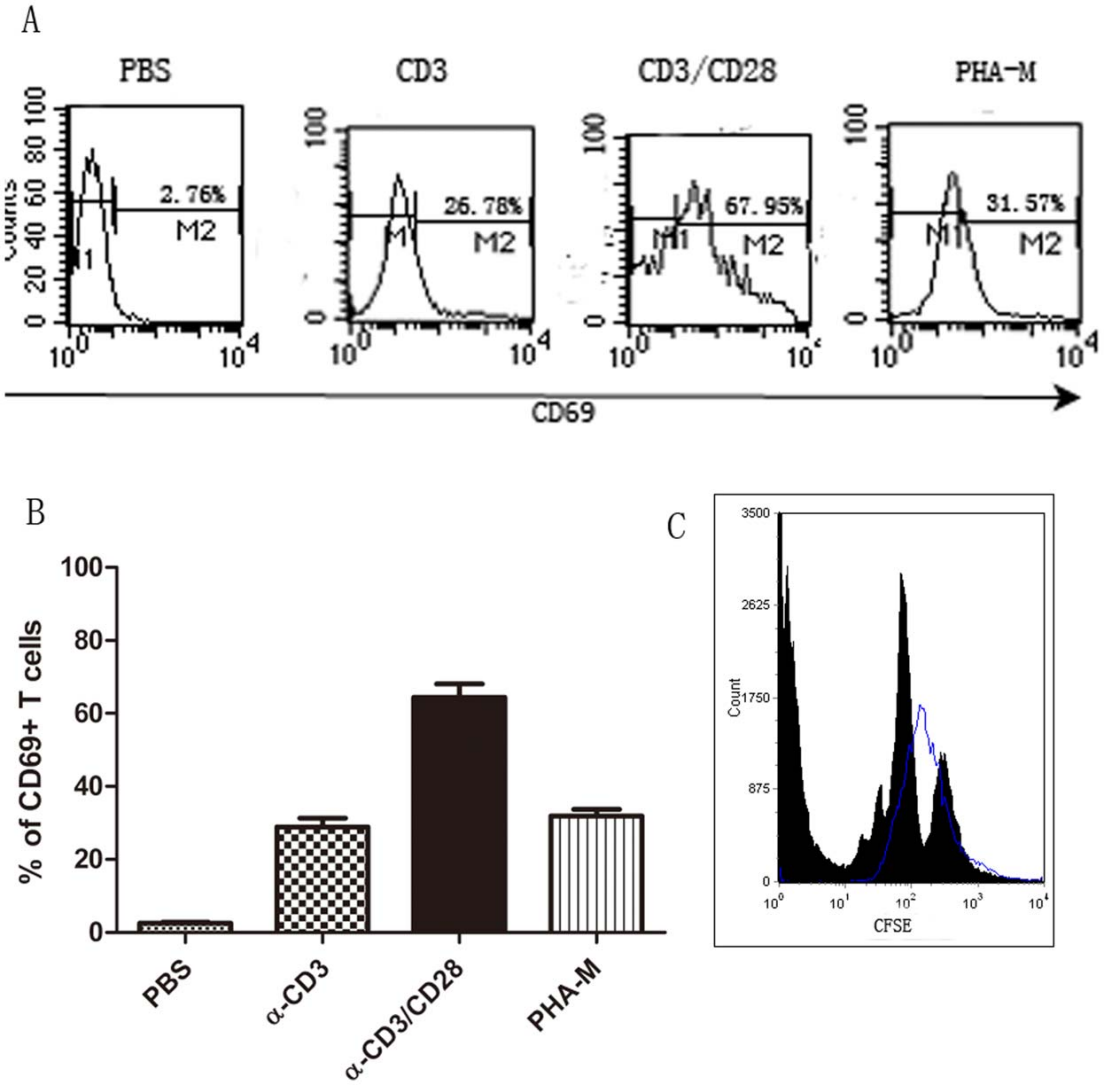
CD4^+^T cells stimulating assay by flow cytometry. **A and B**, Purified naïve CD4^+^T cells were cultured in wells with PBS, anti-CD3 mAb alone (5 µg/ml), anti-CD3 mAb (5 µg/ml), plus anti-CD28 mAb (2 µg/ml), and PHA-M (10 ng/ml) as indicated. After 3 days culturing, the expression of CD69 was assessed by flow cytometry. The percentages represented positive CD69 populations after CD4^+^T stimulation. The histogram of a representative experiment is presented in A (blank line, CD69 staining). CD4^+^T cells stimulated by anti-CD3 Ab plus anti-CD28 Ab elevated CD69 expression by 67.95%, compared with PBS (2.76%), anti-CD3 alone (26.78%) or PHA-M (31.57%), displaying the most efficient T cells proliferation. The summary of 3 independent experiments is presented in B. **C**, Cell division of CD4^+^T cell subpopulations were measured by CFSE dilution on day 3 by flow cytometric analyses of CD3/CD28 stimulating cells. (blue line, Isotype control; blank, CD69 staining).

Before a cytokine level assay, we used anti-CD3 Ab plus anti-CD28 Ab to stimulate naïve CD4^+^T cells proliferation in vitro. T cell proliferation and activation by anti-CD3 alone, anti-CD3 Ab plus anti-CD28 Ab, or PHA-M were detected with the CD69 marker and CFSE. In a representative study, anti-CD3 Ab plus anti-CD28 Ab elevated CD69 expression in CD4^+^T cells by 67.95%, compared with PBS (2.76%), anti-CD3 alone (26.78%) or PHA-M (31.57%) (Fig. 6A). The summary was described in Fig. 6B in three independent experiments. Three days after stimulation, T cell proliferation was measured by CFSE labeling dilution. As demonstrated in Figure 6C, PCR stimulated T cell proliferation in the CD3/CD28 antibody group (represented by multiple peaks), whereas no proliferation was observed in PBS control group (blue line). Thus, we determined that anti-CD3 Ab plus anti-CD28 Ab resulted in efficient T cell proliferation in vitro.

**Figure 7:**
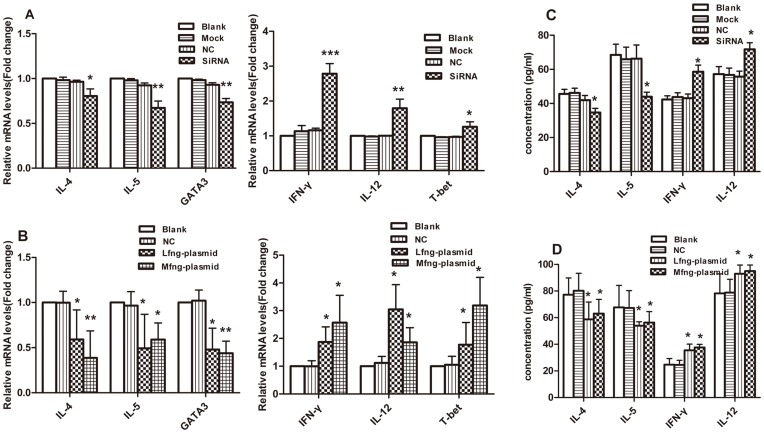
Down-regulation of Rfng and overexpression of Lfng or Mfng decreased their ability to promote Th2 subsets but elevated the ability to promote Th1 subsets. **A**, Real-time PCR analysis was performed to detect the IL-4, IL-5, IFN-γ, IL-12, T-bet, GATA-3 levels in SiRNA interference CD4^+^T cells. Blank-treated results were taken as 1. Results are from three independent experiments. The data for each group are expressed as means±SEM. *p<0.05, **p<0.01, ***p<0.001, significant differences between SiRNA-Rfng group and SiRNA-scramble group (NC), mock control group or blank group. **B**, the IL-4, IL-5, IFN-γ, IL-12, T-bet, GATA-3 mRNA levels of Lfng plasmid group or Mfng plamid group were determined by real-time PCR analysis. *p<0.05, **p<0.01, significant differences between Lfng (Mfng) plasmid group and pEGFP-N1 group (NC) or blank control group. **C and D**, the IL-4, IL-5, IFN-γ, IL-12 concentrations in supernatants were determined by ELISA analysis. The data for each group are expressed as means±SEM. *p<0.05.

Transfected naïve CD4^+^T cells were stimulated by anti-CD3 plus anti-CD28 for 3 days. Q-PCR was performed to detect the mRNA levels of IL-4, IL-5, IFN-γ, IL-12, T-bet, and GATA-3. In the Rfng-siRNA group, the IL-4, IL-5, and GATA-3 mRNA expression levels showed a significant decrease, while the IFN-γ, IL-12, and T-bet mRNA expression levels showed a significant increase compared with the scramble siRNA group, the mock control group or the blank control group (Fig. 7A). The Lfng plasmid group and the Mfng plasmid group demonstrated a decrease in IL-4, IL-5, and GATA-3 mRNA expression but an increase in IFN-γ, IL-12, and T-bet mRNA expression (Fig. 7B).

The concentrations of IL-4, IL-5 IFN-γ, and IL-12 in culture supernatants were detected by ELISA measurement. The siRNA interference group showed significant decreases in IL-4 and IL-5 levels, while the Lfng and Mfng overexpression groups showed increases in IFN-γ and IL-12 levels. (Fig. 7C, D). This proved that down-regulation of Rfng and overexpression of Lfng or Mfng inhibited Th2 subsets but promoted Th1 subsets.

### Lfng, but not Mfng or Rfng, Partly Inhibited Notch Signaling in Asthmatic CD4^+^T Lymphocytes

As shown above, the expression of Fringe was correlated with Th1/Th2 subsets. Fringe acted as one of the Notch signaling modulators, and the role of Notch signaling pathway in peripheral Th cell activation and differentiation has been highlighted recently [30], [31], [32]. We therefore hypothesized that the participation of Fringe regulating Th1/Th2 subsets may be due to its ability to modulate Notch signaling in naïve CD4^+^T cells.

**Figure 8:**
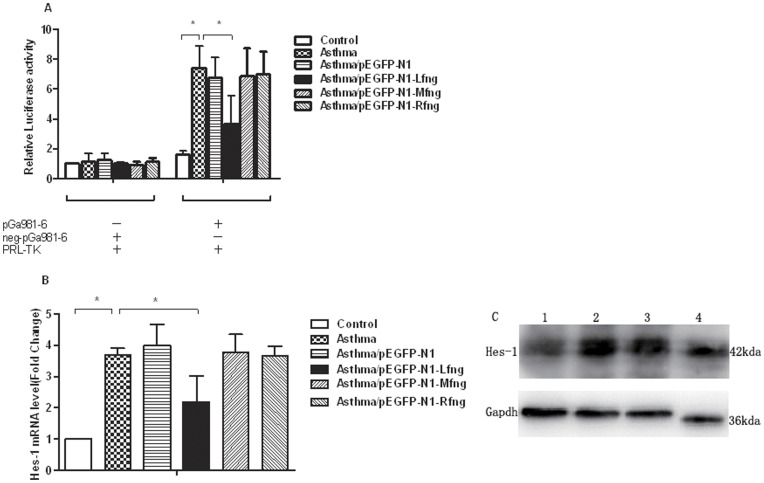
Notch signaling assay in asthmatic naïve CD4^+^T cells. **A**, Asthmatic CD4^+^T cells were transfected with Lfng plasmid and RBPJ-κ luciferase reporter plasmid pGa981-6. Then the luciferase activities were analyzed and normalized to Renilla luciferase activity in control group, asthmatic group, asthmatic CD4^+^T cells treated with control vector pEGFP-N1 and asthmatic CD4^+^T cells treated with Lfng, Mfng or Rfng group. We found that Notch signaling was activated in asthmatic group and overexpression of Lfng led to a reduction in Notch signaling activity**.** But there was no significant differences in Mfng or Rfng group. The results are shown as mean±SEM from three samples. *p<0.05; **p<0.01. The results are from one representative experiment of three independent experiments. **B**, Realtime PCR was performed to detect the mRNA levels of Hes-1. The mRNA levels of control CD4^+^T cells group were taken as 1. Results are from three independent experiments. The data for each group are expressed as means±SEM. *p<0.05, significant differences between asthmatic group with control group, or asthmatic group with asthmatic CD4^+^T cells transfected with Lfng, Mfng or Rfng group. The Hes-1 mRNA decreased in Lfng group comparing with the asthmatic counterparts, but there was no significant differences in Mfng or Rfng group. **C**, Hes-1 protein levels in each group. Lane 1, control CD4^+^T cells; Lane 2, asthmatic CD4^+^T cells; Lane 3, asthmatic CD4^+^T cells transfected with pEGFP-N1 plasmid; Lane 4, asthmatic CD4^+^T cells transfected with Lfng plasmid. Representative of one of three similar experiments. The Hes-1 protein decreased in Lfng treated group.

To test whether Fringe regulates Th differentiation through Notch signaling, we used three distinct assays. First, we modified a Notch-dependent reporter assay which has been previously described to examine the effect of Lfng, Mfng or Rfng on Notch signaling activity in asthmatic CD4^+^T lymphocytes. In this assay, asthmatic or control CD4^+^T lymphocytes were transfected with pGa981-6 and PRL-TK as control vector, along with pEGFP-N1-Lfng, pEGFP-N1-Mfng or Rfng-specific SiRNA respectively. We found that Notch signaling was significantly activated in the asthmatic group compared with the control group. Overexpression of Lfng led to a reduction in Notch signaling activity, but there were no significant differences in Mfng overexpression and Rfng-SiRNA interference group (Fig. 8A).

In a second approach to test the Notch signaling activity, we analyzed the endogenous expression of Hes-1 gene, which was a downstream target gene of Notch signaling. Real-time PCR was performed to detect the Hes-1 mRNA. The Hes-1 mRNA levels showed a significant increase in the asthmatic group compared with control group. Overexpression of Lfng down-regulated the Hes-1 mRNA compared with the asthmatic group, while there was no significant differences in Mfng or Rfng interference group (Fig. 8B).

Third and final, Hes-1 protein levels were analyzed by Western blot. The protein levels of each group were consistent with their respective mRNA levels. The Hes-1 protein decreased in Lfng overexpression group (Fig. 8C), but there was no significant differences in Mfng or Rfng treated group (data not shown). The outcomes proved that Notch signaling was activated in the asthmatic group and that overexpression of Lfng, but not overexpression of Mfng or Rfng knockdown inhibited the activation of Notch signaling in asthmatic CD4^+^T cells to some extent.

### Overexpression of Lfng on Asthmatic CD4^+^T Lymphocytes Promoted Notch-independent Th1 Cytokine Responses and Inhibited Th2 Cytokine Responses via a Notch-dependent Mechanism

We found that Lfng promoted Th1 cytokine while simultaneously inhibiting Th2 cytokine and Lfng attenuated Notch signaling activation as described above. We next asked whether the observed effects of Lfng on naïve CD4^+^T cell differentiation are Notch signaling dependent.

**Figure 9:**
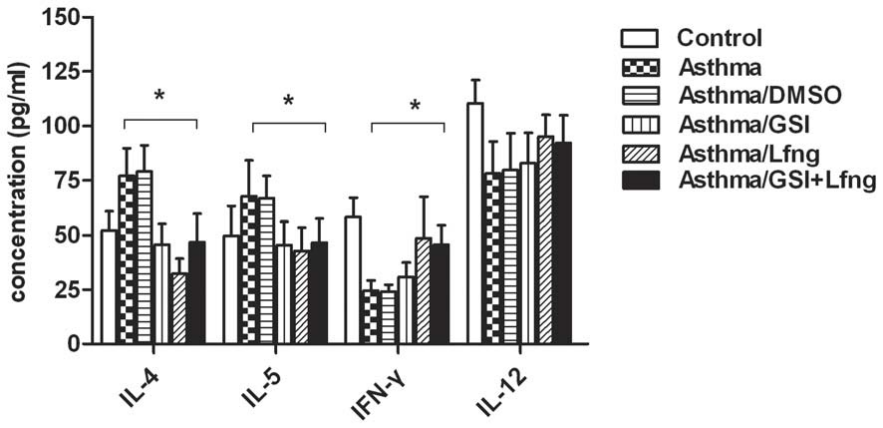
Cytokine production from asthmatic naïve CD4^+^T cells pretreated with or without GSI. CD4^+^T cells pretreated with or without GSI and transfected with Lfng cDNA. All the groups, including control group, asthmatic group, asthmatic/DMSO (asthmatic CD4^+^T treated with DMSO as negative control), asthmatic/GSI (asthmatic CD4^+^T treated with GSI), asthmatic/Lfng (asthmatic CD4^+^T treated with Lfng vector) and asthmatic/GSI+Lfng (asthmatic CD4^+^T treated with GSI and Lfng vector) cells were stimulated by anti-CD3/anti-CD28 antibody and cultured for 3 days. The culture supernatants were collected to detect the IL-4, IL-5, IFN-γ, IL-12 levels by ELISA. *p<0.05. The results are from one representative experiment of three independent experiments. Lfng overexpression almost had the same effect as GSI blockage on Th2 cytokine promotion (IL-4, IL-5) but had a greater effect on Th1 cytokines (IFN-γ, IL-12) than GSI treatment.

We reasoned that if the regulating effects of Lfng on Notch were independent, then blockage of Notch signaling would have no effect, whereas if the effects were dependent, then the administration of Lfng overexpression would diminish, even vanish. Lfng vector trasfected into naïve CD4^+^T cells of asthmatic group as described above. CD4^+^T cells pretreated with GSI or DMSO as negative control. All groups were subsequently restimulated with anti-CD3 and anti-CD28 (2 µg/ml, respectively) for an additional 3 days. Then the cytokine levels were detected in all the groups. The GSI pretreatment markedly reduced Th2 cytokine production, which was consistent with the Lfng-treated group, but enhanced Th1 cytokine production slightly compared to asthmatic CD4^+^T cells. The asthma/GSI group treated with Lfng did not show an additive effect compared with cells pretreated with GSI or treated with Lfng alone (Fig. 9), which meant that Lfng overexpression almost had the same effect as GSI blockage on Th2 cytokine promotion but had a greater effect on Th1 cytokines than GSI treatment. The regulating effect of Lfng on Th2 differentiation was Notch-dependent while the effect of Th1 differentiation did not depend on Notch signaling thoroughly. Maybe Lfng overexpression promoted Th1 differentiation via a Notch-independent pathway. These data indicated that Lfng up-regulated had two distinct but complementary effects on CD4^+^T cell differentiation: the promotion of Th1 cells development and the inhibition of Th2 cell development, both of which leading to polarized Th1 responses.

## Discussion

In this article, we present an OVA-sensitized and -challenged rat model of allergic asthma characterized by pulmonary eosinophilia and airway hyperresponsiveness. Asthmatic CD4^+^T cells producing Th2 cytokines were observed in the BAL fluid and serum. Several studies have shown that CD4^+^T cells and Th2 cytokines play pivotal roles in the development of allergic asthma [1], [33], [34]. Previous reports have emphasized the vital role of Notch signaling in T helper cell maturation and differentiation. However, the events that trigger Th cell differentiation remain poorly understood, especially regarding the development of allergic asthma. Fringe, acting as a modulator of the Notch extracellular domain, regulates Notch receptor-ligand combinations and, subsequently, downstream transcription. We hypothesized that Fringe may have influential effects on Notch signaling and CD4^+^T cells differentiation. Several studies have researched Notch signaling in Th cell differentiation, but the role of Fringe has rarely been considered. We proposed to explore the relationship between Fringe and CD4^+^T cells differentiation regarding the pathogenesis of allergic asthma.

In the present study, Lunatic Fringe (Lfng) and Manic Fringe (Mfng) were found to be down-regulated whereas Radical Fringe (Rfng) was up-regulated in naïve CD4^+^T cells of asthmatic group. The levels of gene expression were paralleled by increases or decreases in three Fringe homolog protein levels. To investigate the role of the three Fringe homologs on CD4^+^T cells in Th cell differentiation, two approaches were used: the silencing of Rfng expression and the overexpression of Lfng and Mfng in naïve CD4^+^T cells. Both Rfng gene down-regulation and Lfng and Mfng up-regulation were achieved and developed a higher concentration of Th1 cytokines and a lower concentration Th2 cytokines compared with untreated CD4^+^T cells. The results indicated that the three Fringe homologs could regulate Th cell differentiation. Despite this, very little is known about how Fringes control Notch-dependent development or whether a Notch-independent pathway exists.

The modification of Notch by Fringe controls the growth, patterning, and compartmentalization of the wing in fruit flies [25]. Lfng can mediate T cell maturation and lung alveogenesis through Notch signaling [28], [35], [36]. Fringe can modulate different sites of Notch receptor and show functional diversity [37], [38]. Gelfand and colleagues demonstrated that the down-regulation of Jagged1 in BMDCs and Notch signaling in CD4^+^T cells resulted in decreased Th2 cytokines and Th2 polarization in vivo [29]. Nonetheless, few studies have focused on the function of Fringe regulating Th cell differentiation, especially regarding allergic asthma. We explored the Th cell differentiation regulated by Fringe in asthmatic rat models via several approaches, such as receptor-ligand binding, Notch signaling activation, and downstream target gene transcription.

In principle, there are three methods for Notch signaling modulating: regulating receptor-ligand combinations, influencing Notch proteolysis, and triggering intracellular trafficking and downstream transcription [39]. Fringe has been reported to regulate receptor-ligand binding at the cell surface of T cells or BMDCs, which activates Notch signaling [21], [22]. To focus on Notch signaling, we next attempted to evaluate the activation of Notch signaling in asthmatic CD4^+^T cells via an inhibitor of Notch signaling, γ-secretase inhibitor (GSI), and the expression of downstream target genes. We also compared the effects associated with Fringe interference. Our data showed that Lfng, but not Mfng or Rfng, partly inhibited Notch signaling in asthmatic naïve CD4^+^T cells. So we emphasized Lfng function without the other two Fringe homologs and performed experiments in vitro through cell cocultivation and a cytokine assay. We found that asthmatic CD4^+^T cells treated with Lfng overexpression showed a depressed Notch signaling activity comparing with the asthmatic counterparts and naïve CD4^+^T cell differentiated into Th1 cells rather than Th2 cells. We hypothesized that Fringe regulated T-helper cell differentiation via its effect on Notch signaling rather than a Notch-independent mechanism. The assay proved that Lfng overexpression almost had the same effect as GSI blockage on Th2 cytokine promotion but had a greater effect on Th1 cytokines than GSI treatment. Taken together, the above findings are consistent with the ideas that Fringe can transfer Th2 into Th1 polarization in allergic asthma through Notch signaling.

A large number of reports have illustrated that Notch signaling promotes either Th1 or Th2 differentiation [14], [31], [39], [40]. Some groups emphasize the role of Notch in Th2 polarization, while others focus on Th1. In loss of function experiments, the absence of Notch signaling markedly diminished Gata3 expression and, subsequently, Th2 cell responses [30], [41]. Using dominant negative MAML transgenic mice, Pear and colleagues showed that Notch signaling was required only for Th2 cell responses [40]. In contrast, γ-secretase inhibitor prevented Th1 polarization in vivo and in vitro through down-regulation of Tbx21 [15]. No consensus exists about the role of Notch in T helper differentiation. Using Notch1-targeted siRNA, our team has found that Notch signaling plays a vital role during CD4^+^T cells activation in the asthmatic pathomechanism [36].

In mammals, there are four Notch receptors, five Notch ligands and three Fringe homologs that are expressed on T cells or APCs. The fact that different Notch ligands and receptors can, in different settings, elicit apparently contradictory responses suggests that Notch signaling is more complex and controversial [36]. Recent evidence has indicated that Delta1 promotes Th1 responses and that Jagged1 promotes Th2 responses [13], [14], [42].

Our data are supportive of this idea, as shown in our Lfng experiments above, Notch signaling is more important for Th2 differentiation. We demonstrate that blocking of Notch signaling leads to diminished Th2 cells differentiation but a slight increased Th1 cells differentiation. Lfng can still increase Th1 cytokine production a lot after the blocking of Notch signaling, proving a Notch-independent mechanism. We presumed that the low expression of Lfng in asthmatic naïve CD4^+^T cells can not inhibit Notch signaling activation, leading to strikingly Th2 cytokine production and a relative diminished Th1 cytokine production. This would suggest therapeutic approaches that Lfng may benefit diseases associated with excessive production of Th2 cytokine, such as asthma.

Regarding the complex internal environment, the regulation of naïve CD4^+^T cells differentiation by Notch in vivo, especially in allergic asthma, must be extraordinary profound. In addition, the experiments on the other two Fringe homologs and downstream regulation of Notch are not well designed. We will seek to improve these experiments in the future.

Most studies highlight Notch signaling in Th cell development and differentiation in the context of fundamental research. Our studies demonstrate the function of Fringe in clinical diseases, which has seldom been done. Fringe may have two linked effects: the inhibition of Th2 cell development and the promotion of Th1 cell development. Understanding the mechanisms underlying the suppression of Th2 cell differentiation and the promotion of Th1 cell development by Fringe may result in new therapeutic targets for treating asthma, allergies, and other Th2-mediated pathologies.

## Supporting Information

Figure S1 **The purification of naïve CD4+T cells were detected by flow cytometry.** Naïve CD4+T cells were stained with antibody CD3, CD4, CD25, CD69, CD45RA and CD45RO and follow by flow cytometry analysis. All the naïve CD4+T cells expressed high levels of CD3, CD4 and CD45RA, but scarcely express CD25, CD69 and CD45RO. The purity of naïve CD4+T cells population consistently >90%.
(TIF)
