# Supplementary figures and images for: Fringe Controls Naïve CD4+T Cells Differentiation through Modulating Notch Signaling in Asthmatic Rat Models
Source: PLoS One. 2012 Oct 10;7(10):e47288. doi: 10.1371/journal.pone.0047288 (PMC3468515; doi:10.1371/journal.pone.0047288)

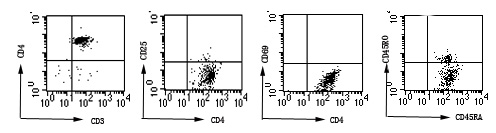

Supplement: Figure S1 — The purification of naïve CD4+T cells were detected by flow cytometry. Naïve CD4+T cells were stained with antibody CD3, CD4, CD25, CD69, CD45RA and CD45RO and follow by flow cytometry analysis. All the naïve CD4+T cells expressed high levels of CD3, CD4 and CD45RA, but scarcely express CD25, CD69 and CD45RO. The purity of naïve CD4+T cells population consistently >90%. (TIF) [file pone.0047288.s001.tif]
